# Small-Scale Production of *Amblyseius tamatavensis* with *Thyreophagus cracentiseta* (Acari: Phytoseiidae, Acaridae)

**DOI:** 10.3390/insects12100848

**Published:** 2021-09-22

**Authors:** Marcela Massaro, Matheus Montrazi, José Wagner S. Melo, Gilberto J. de Moraes

**Affiliations:** 1 Departamento de Entomologia and Acarologia, Universidade de São Paulo, Piracicaba 13418-900, Brazil; mmontrazi@outlook.com (M.M.); moraesg@usp.br (G.J.d.M.); 2 Departamento de Fitotecnia, Universidade Federal do Ceará, Fortaleza 60020-181, Brazil; wagnermelo@hotmail.com

**Keywords:** biological control, predatory mite, factitious food, astigmatina

## Abstract

**Simple Summary:**

*Amblyseius tamatavensis* is a predatory mite that can be used to control the whitefly *Bemisia tabaci*. This predator can be maintained under laboratory conditions when fed with pollen of *Typha domingensis* or the astigmatine mites *Thyreophagus cracentiseta* or *Aleuroglyphus ovatus*. The aim of this work was to compare the rates of production of *A. tamatavensis* in closed units containing *T. cracentiseta* as food, at different combinations of numbers of predator inoculated, periods of production and volumes of rearing units. The first results showed that final predator densities increased with increasing production periods up to 30–45 days, reducing afterward. Likewise, maximum final densities in each unit increased with increasing predator inoculation levels, up to 200 predators per rearing unit. The results led us to select the proportion of 150 predators per unit for a production period of 30 days to evaluate the effect of the size of the experimental unit. Then, in the second part of the study, a direct relationship was observed between volume and final predator density. Hence, it can be concluded that progressively larger numbers of predators can be obtained with progressively larger rearing units.

**Abstract:**

*Amblyseius tamatavensis*, a predatory mite, has been mentioned as potentially useful for the control of *Bemisia tabaci*. The objective of this work was to compare the production rates of *A. tamatavensis* in closed units containing *T. cracentiseta* as food, at different combinations of numbers of predator inoculated, periods of production and volumes of rearing units. Final predator densities increased with increasing production periods up to 30–45 days, reducing afterward. Likewise, maximum final densities increased with increasing predator inoculation levels, up to 200 predators per rearing unit. The results led us to select the proportion of 150 predators per unit for a production period of 30 days to evaluate the effect of the size of the experimental unit. Then, in the second part of the study, a direct relationship was observed between volume and final predator density (y = 8610.25x + 2166.04; R^2^ = 0.99; *p* < 0.0001). It was also calculated that r_i_ value was quite stable (0113–0.119), with a calculated value of 0.115 at all volumes of rearing units. It can be concluded that progressively larger numbers of predators can be obtained with progressively larger rearing units.

## 1. Introduction

The excessive use of chemicals for pest control in intensive agricultural production can cause environmental contamination and other pest problems [1]. An alternative is augmentative releases of biological control agents, providing they can be produced at affordable prices.

Some mite and small insect pests (as fungus gnats, thrips and whitheflies) have been controlled with the use of predatory mites [2]. Several methods have been evaluated for the production of these control agents, especially those of the families Phytoseiidae and Laelapidae [2]. The production of some predatory mites involves the use of phytophagous mites as food [3], but others can be produced more easily with the use of factitious food, especially mites of the suborder Astigmatina of the order Oribatida [4]. Most often, astigmatine mites used as factitious food can be produced on stored food items, commonly their preferred substrates [5]. These mites can then be offered to the predators in closed containers [4,6]. This process is relatively cheap, facilitating the use of biological control by growers. Despite worries about the possibility that the use of factitious food could result in the production of biological control agents less effective in the field, this had not been demonstrated, and thus astigmatine has been widely used in predatory mite production [7].

Worldwide, the whitefly *Bemisia tabaci* (Gennadius) is a major pest of several plant species [8], including high value horticultural crops. This pest has been difficult to control because of its resistance to registered pesticides. The phytoseiid *Amblyseius swirskii* Athias-Henriot has been extensively used in different countries for whitefly control [9,10], while *Amblyseius tamatavensis* Blommers has been mentioned as a potential candidate predator for the same purpose [11]. This predator has been recently registered in Brazil for that purpose.

*Amblyseius tamatavensis* is known to occur naturally in several tropical and subtropical regions of the world [12]. This predator can be reared in the laboratory with the use of a technique described by McMurtry and Scriven [13], when fed with pollen of *Typha domingensis* Persoon or on the astigmatine (Acaridae) *Aleuroglyphus ovatus* (Troupeau) and *Thyreophagus cracentiseta* Barbosa, O’Connor & Moraes (Acaridae) [11,14]. *Tyreophagus cracentiseta* has been used for the maintenance of predatory mites in our laboratory for years, without indication of undesirable effects on humans. Massaro [14] reported that when fed with *T. cracentiseta*, the daily oviposition rate of *A. tamatavensis* was about as high as reported by Cavalcante [11] on *A. ovatus*, and much higher than also reported by Cavalcante [11] on pollen of *T. domingensis*.

The objective of this work was to compare the production rates of *A. tamatavensis* in closed units containing *T. cracentiseta* as food, at different combinations of numbers of predators inoculated, periods of production and volumes of rearing units.

## 2. Materials and Methods

*Thyreophagus cracentiseta* and *A. tamatavensis* were obtained from laboratory colonies initiated with specimens collected at ESALQ/USP (Piracicaba, Brazil) and maintained on units similar to those described respectively by Freire and Moraes [15] and McMurtry and Scriven [13]. The first was fed with a mixture of yeast and wheat bran, and the second, with *T*. *domingensis* pollen and all development stages of *T. cracentiseta*.

### 2.1. Predator Inoculation Density × Production Period

**Figure 1:**
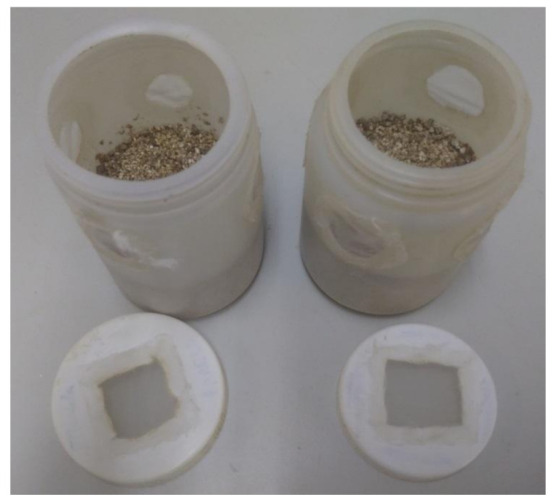
Rearing units (plastic cylinders 12 cm high × 7.5 cm in diameter, volume ca. 500 mL) with five holes in the wall of the upper half, closed with polyester mesh of 0.2 mm openings, to allow ventilation. Each unit was half filled with vermiculite.

Each rearing unit consisted of a plastic cylinder (about 12 cm high × 7.5 cm in diameter; volume ca. 500 mL) with five holes in the wall of the upper half, closed with polyester mesh of 0.2 mm openings, to allow ventilation (Figure 1). Each unit was half filled with vermiculite (maintained in the previous 24 h at 70 °C to kill possible contaminant mites) to increase surface area, promoting better distribution of the mites.

The vermiculite was humidified with 3 mL of distilled water, and then either 50, 100, 150, 200, 250, 300, 350 or 400 adult predators were introduced onto each unit. Once a week, predators were fed with all stages of *T*. *cracentiseta* by adding to each unit 200 mg of the rearing substrate containing the mites, taken from the stock colony. The density of all active stages of *T. cracentiseta* was estimated by dispersing a sample of 200 mg of the substrate in 100 mL of water, and then taking 10 subsamples of 1 mL for estimating the number of mites using a Peter´s counting slide, regularly employed in nematology. The material was shaken vigorously before taking each subsample, to homogenize the distribution of the mites in the liquid. This evaluation was conducted three times. The estimated density was approximately 38,000 eggs and 80,000 mites of other stages per 200 mg of the substrate. These numbers are considered compatible with predator predation capacity, allowing a surplus amount of prey even at the highest rate of predator inoculation (400 predators). This was checked in a parallel test, when each adult female *A. tamatavensis* was estimated to prey daily on 6.9 ± 0.2 eggs, 16.6 ± 0.2 post-embryonic immatures or 11.8 ± 0.3 adults of *T. cracentiseta* (*n* = 50 predators/prey age class).

Once a week, the same amounts of distilled water and food were introduced to each unit, which was then gently rotated, to speed mite dispersion. The units were maintained for 15, 30 or 60 days in a rearing chamber, in darkness. Temperature and humidity within one of the units was measured every 60 min with a data logger (Instrutherm HT-500); averages for the duration of the study were respectively 25 ± 1 °C and 70 ± 10% RH. Two rearing units were subjected to each combination of initial number of predators and duration of production period.

The total number of all active stages of the predator in each unit at the end of respective production period was evaluated by processing its content in a modified Berlese-Tullgren funnel for 5 days, collecting the mites in a flask with 50 mL of 70% ethanol. After extraction, the volume in the flask was completed to 100 mL with 70% ethanol, and predators were quantified as previously described in this section. With these numbers, the respective instantaneous rates of reproduction (r_i_) were calculated, using the formula provided by Hall (1964) [16]: r_i_ = ln (n_f_/n_o_)/∆t, where: n_o_ inoculation density, n_f_: final density and ∆t: period of production.

### 2.2. Volume of the Rearing Unit

In the second part of the work, the performance of the predator was evaluated at different volumes of rearing units. The proportion between the amount of food provided and the number of inoculated predators was maintained constant at all volumes. The proportion selected for use in this part of the work was that corresponding to the combination providing the highest r_i_ values at the lowest possible number of inoculated predators in the first part of the work. Each treatment (volume of the unit) was evaluated in duplicate.

### 2.3. Statistical Analyses

In evaluating the interaction between predator inoculation density and period of production, for each rate of predator inoculation (x), data were subjected to regression analyses, relating either final predator density or instantaneous rate of reproduction (y) in each unit with time of predator production. Regressions were obtained using “curve-fitting” procedure of TableCurve 2D (Systat, San Jose, CA, USA). Among the significant models (*p* < 0.05), a single one was selected to be used for each rate of predator inoculation. The selection was based on simplicity, parsimony and high F and R^2^ values. Residual values were also taken into account for each analysis to validate parametric premises.

In evaluating the effect of the volume of the rearing unit, the same type of analysis used in the previous section was conducted, relating final predator densities or instantaneous rates of reproduction (y) with volume of the rearing unit (x).

## 3. Results

### 3.1. Predator Inoculation Density × Production Period

**Figure 2:**
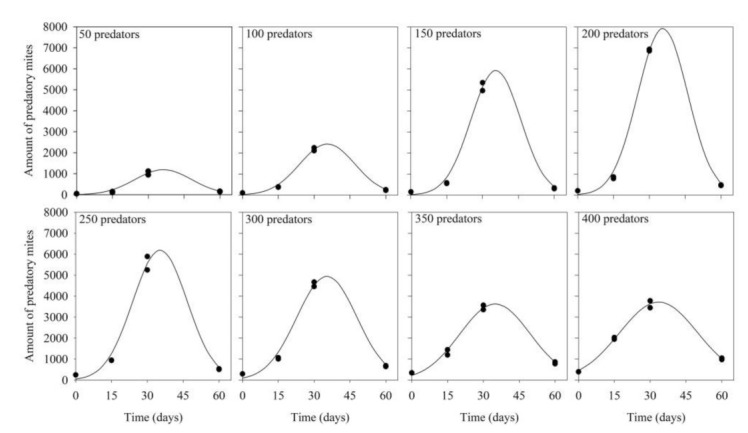
Density of *Amblyseius tamatavensis* in each 500 mL container at different production periods on *Thyreophagus cracentiseta*, for different levels of predator inoculation (25 ± 1 °C, 70 ± 10% RH, in the darkness).

Similar patterns of variation of the numbers of predators with increasing production periods were obtained at all predator inoculation densities (Table 1; Figure 2; R^2^ ≥ 0.96; *p* < 0.05). Densities increased with increasing production periods, up to 30–45 days, reducing afterward. Likewise, maximum densities increased with increasing predator inoculation levels, up to 200 predators per rearing unit (when the final estimated number was almost 8000 predators), reducing afterward.

**Figure 3:**
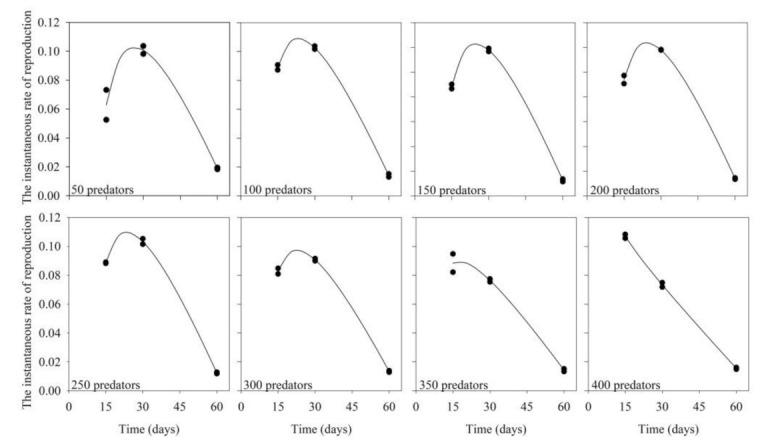
Instantaneous rate of reproduction of the predator *Amblyseius tamatavensis* produced in each 500 mL containers at different production periods on *Thyreophagus cracentiseta*, for different levels of predator inoculation (25 ± 1 °C, 70 ± 10% RH, in the darkness).

Variation of the maximum values of instantaneous rate of reproduction (r_i_) was small (0.09–0.12) over the evaluated predator inoculation rates (Table 2; Figure 3; R^2^ ≥ 0.96; *p* < 0.05). Calculated r_i_ was highest at 30 days of production or at a slightly shorter period, reducing afterward. The exceptions occurred with inoculations of 350 and 400 predators, when the highest r_i_ values occurred at 15 days of production. The highest r_i_ value at the lowest possible number of inoculated predators was recorded when 150 predators were inoculated and the production unit was maintained for a period of 30 days (Figure 3).

### 3.2. Volume of the Rearing Unit

Taking into account the highest r_i_ and the lowest predator inoculation generating that rate (production unit maintained for 30 days after inoculation of 150 predators per 500 mL unit), the proportion between the amount of food provided and the number of inoculated predators was calculated [(76 eggs and 160 post-embryonic stages of *T. cracentiseta*): (0.3 *A. tamatavensis*)]/1 mL, to be maintained at all evaluated volumes.

**Figure 4:**
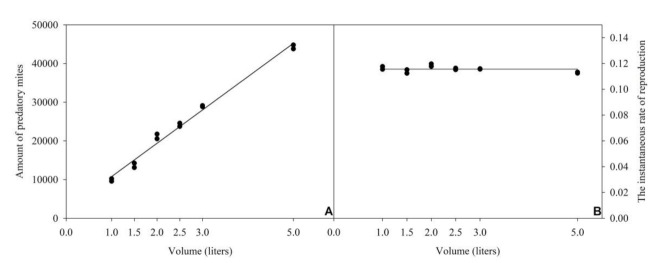
Relation between final density of *Amblyseius tamatavensis* (**A**) or instantaneous rate of reproduction (**B**) and volumes of containers, using a constant proportion between the amount of food provided and the number of inoculated predators (76 eggs and 160 post-embryonic stages of *Thyreophagus cracentiseta*: 0.3 *A. tamatavensis*/mL), for 30 days of production, at 25 ± 1 °C, 70 ± 10% RH, in the darkness.

By doing that, a direct and positive relationship was observed between final predator density and volume (y = 8610.25x + 2166.04; R^2^ = 0.99; *p* < 0.0001) (Figure 4A). Obtained r_i_ values were rather similar (0.113–0.119) at all evaluated volumes (Figure 4B), so that it was estimated as constant (y = 0.115) within the range of evaluated volumes.

## 4. Discussion

In the first part of the study, to determine the effect of rate of predator inoculation and production period, determination of maximum r_i_ values at the shortest production period for the two highest predator inoculation rates could be a function of competition among predators for the fixed amount of prey offered in each unit or for space. However, competition for food was probably not a major factor in this study, given the surplus number of prey (not counted) observed while determining predator densities. Hence, it seems that most important could have been competition for space.

However, the reduction of the final predator densities at the longest period of production evaluated in this work, even at the lowest predator inoculation rates, suggests that other factors in addition to competition should be involved. At the lowest rate of predator inoculation, it seems that an important factor could be the excessive number of prey available in each unit. Without taking into account the reproduction of the prey, at the very lowest rate (50 predators), each predator had an average of 760 eggs and 1600 post-embryonic stages available for consumption in the first day of the study. That high number could have disturbed the predators, reducing their biological performance. However, probably also important could be the accumulation of organic material (excess of prey food, mite feces, metabolic waste products, and dead prey and predators) and volatiles resulting from its decomposition in the rearing unit.

Although food provision to the predators was done once a week, the total volume of substrate (vermiculite and astigmatine food) in each unit increased very slightly during the study. However, the addition of food made the substrate seemingly more compact, for the penetration of part of the astigmatine mite food into the open spaces of the vermiculite layer, probably also making it physically less suitable to the predator.

The findings of the present study may serve as a basis for the small-scale production of *A. tamatavensis* (and probably of other predators that can feed on astigmatine mites), making possible its practical use in small farming areas. Cavalcante et al. [17] reported promising results in a trial to control *B. tabaci* when 15 *A. tamatavensis* were released per bell pepper plant. Commercial cultivation of this crop is usually done at a rate of four plants per m^2^. Hence, it could be concluded that the predators produced in a 5-L container (which potentially produces an average of 40,000 mites in 30 days) could be sufficient for release over an area of some hundred m^2^.

As shown in the second part of this study, progressively larger numbers of predators can be obtained with progressively larger rearing units. Thus, an upscaled similar system could conceivably be adapted for larger scale production of this predator. Although predatory mites can often be reared on plants infested with phytophagous mites in greenhouses, their production with the use of astigmatines is usually considered much less expensive [6], comparing the requirements for infrastructure (mainly) and other production costs of both systems. Despite the concerns raised by Pijnakker et al. [7] about the use of astigmatine mites to maintain predatory mites on plants, the production of *T. cracentiseta* and its use for trials with those predators in the laboratory has not shown any type of undesirable side effect.

Complementary studies should be conducted to optimize the evaluated production system. Factors to be evaluated include: (a) use of different types of porous filling material replacing or in combination with vermiculite for increasing surface area; (b) different rates of prey provision to prevent presence of excessive or insufficient food; (c) promotion of forced air current in the rearing units to remove volatile contaminants; (d) use of larger rearing containers; (e) the efficiency of predators produced with this system in comparison with predators produced with other food items. As another species of this same genus (*Thyreophagous entomophagus* (Laboulbène & Robin)) has been reported to cause anaphylaxis when ingested, as summarized by Barbosa et al. [18], it seems advisable that a risk analysis be performed before implementing the large-scale use of *T. cracentiseta* as factitious food.

## Figures and Tables

**Table 1:** Regressions between numbers of the predator *Amblyseius tamatavensis* produced in 500 mL containers and production periods on *Thyreophagus cracentiseta*, for different levels of predator inoculation (*n* = 8 containers per predator level).

Levels of Preda- tor Inoculation	Equation (lny = a + bx + cx^2^)			F Value	R^2^	*p-*Value
	A	B	C			
50	2.41	0.25	−0.003	74.47	0.96	0.00019
100	3.08	0.26	−0.003	259.57	0.99	0.00001
150	2.86	0.32	−0.004	316.88	0.99	0.00001
200	3.20	0.32	−0.004	529.79	0.99	<0.00001
250	3.93	0.27	−0.003	186.51	0.98	0.00002
300	4.56	0.22	−0.003	193.10	0.98	0.00002
350	5.39	0.16	−0.002	214.83	0.98	0.00001
400	6.12	0.12	−0.001	424.77	0.99	<0.00001

**Table 2:** Regressions between instantaneous rate of increase of the predator *Amblyseius tamatavensis* and its maintainment times on *Thyreophagus cracentiseta*, by considering different levels of predator inoculation (*n* = 6 containers per predator inoculation level).

Levels of Predator Inoculation	Equation (y = a + bx + c/x)			F Value	R^2^	*p-*Value
	a	B	c			
50	0.34	−0.004	−3.15	44.33	0.96	0.00592
100	0.30	−0.004	2.32	1241.12	0.99	0.00004
150	0.38	−0.005	−3.28	1558.45	0.99	0.00003
200	0.37	−0.005	−3.04	814.67	0.99	0.00008
250	0.31	−0.004	−2.41	1820.76	0.99	0.00002
300	0.26	−0.003	−1.86	1177.54	0.99	0.00005
350	0.17	−0.002	−0.76	112.73	0.98	0.0015
400	0.12	−0.0018	0.18	1400.07	0.99	0.00004

## Data Availability

The data presented in this study are available in the article.
